# Supplementary material for: A dataset of synthetic, maturation-informed magnetic resonance images of the human fetal brain
Source: Sci Data. 2025 Apr 10;12:602. doi: 10.1038/s41597-025-04926-9 (PMC11986055; doi:10.1038/s41597-025-04926-9)
Supplement: Supplementary file 1 — Supplementary file [file 41597_2025_4926_MOESM1_ESM.pdf]

Supplementary Information
“A dataset of synthetic, maturation-informed magnetic resonance images of the human fetal brain”

Hélène Lajous[1,2,*], Andrés le Boeuf Fló[1,2,3], Pedro M. Gordaliza[2,1], Oscar Esteban[1], Ferran Marqués[3], Vincent Dunet[1], Mériam Koob[1], and Meritxell Bach Cuadra[2,1]
[1]Department of Radiology, Lausanne University Hospital and University of Lausanne, Lausanne, Switzerland
[2]CIBM Center for Biomedical Imaging, Switzerland
[3]Department of Signal Theory and Communications, Universitat Politècnica de Catalunya, Barcelona, Spain
*corresponding author: Hélène Lajous (helene.lajous@unil.ch)

The list below indicates the corresponding **identifiers of the cases simulated in our cohort**. Original cohorts are FeTA data set[1] and its derivatives[2].

	Id	Condition	Gestational age (weeks)	Technical Validation	Reuse potential (training=1)
1	sub-041	Neurotypical	34.8	1	1
2	sub-042	Pathological	23.6	1	0
3	sub-043	Pathological	22.9	1	0
4	sub-044	Neurotypical	27.9	0	1
5	sub-045	Neurotypical	24.7	1	0
6	sub-046	Neurotypical	23.9	1	1
7	sub-047	Pathological	28.1	0	1
8	sub-048	Pathological	27.9	1	1
9	sub-049	Pathological	31.1	1	0
10	sub-050	Pathological	33.1	1	1
11	sub-051	Neurotypical	29.6	0	1
12	sub-052	Pathological	21.2	0	1
13	sub-053	Neurotypical	30.3	0	1
14	sub-055	Pathological	27.1	1	1
15	sub-056	Pathological	26.6	1	1
16	sub-057	Neurotypical	28.2	1	1
17	sub-058	Neurotypical	29.2	0	1
18	sub-059	Neurotypical	34.8	0	1
19	sub-060	Neurotypical	31.7	0	1
20	sub-061	Neurotypical	33	1	1
21	sub-062	Neurotypical	24.4	1	1
22	sub-063	Pathological	21.7	0	1
23	sub-064	Pathological	27.8	0	1
24	sub-065	Pathological	20.9	1	0
25	sub-066	Pathological	21.8	1	1
26	sub-067	Pathological	29	1	1
27	sub-068	Neurotypical	31.5	0	1
28	sub-069	Pathological	27.4	0	1
29	sub-070	Pathological	20.1	1	1
30	sub-071	Pathological	22.4	0	1
31	sub-072	Neurotypical	25.9	1	0
32	sub-073	Pathological	27.2	0	1
33	sub-074	Pathological	23.3	1	1
34	sub-075	Pathological	29	0	1
35	sub-076	Neurotypical	23.2	1	1
36	sub-077	Pathological	26.9	0	1
37	sub-078	Pathological	24	0	1
38	sub-079	Neurotypical	29.1	1	0
39	sub-080	Pathological	26.9	0	1
40	sub-feta002	Pathological	28.2	0	1
41	sub-feta003	Pathological	27.4	0	1
42	sub-feta004	Pathological	25.5	0	1
43	sub-feta005	Pathological	22.6	0	1
44	sub-feta008	Pathological	25.2	0	1
45	sub-feta010	Pathological	27.3	0	1
46	sub-feta012	Pathological	25.9	0	1
47	sub-feta013	Pathological	27.5	0	1
48	sub-feta014	Pathological	26.7	0	1
49	sub-feta016	Pathological	23.3	0	1
50	sub-feta017	Pathological	22.8	0	1
51	sub-feta018	Pathological	28.5	0	1
52	sub-feta019	Pathological	29.2	0	1
53	sub-feta020	Pathological	25.8	0	1
54	sub-feta021	Pathological	26.1	0	1
55	sub-feta022	Pathological	20	0	1
56	sub-feta023	Pathological	23.7	0	1
57	sub-feta024	Pathological	30.4	0	1
58	sub-feta025	Pathological	24.2	0	1
59	sub-feta026	Neurotypical	27.8	0	1
60	sub-feta027	Neurotypical	26.5	0	1
61	sub-feta028	Neurotypical	31.1	0	1
62	sub-feta030	Neurotypical	33.4	1	1
63	sub-feta031	Neurotypical	31.4	1	1
64	sub-feta032	Neurotypical	32.3	0	1
65	sub-feta033	Neurotypical	30	1	0
66	sub-feta035	Neurotypical	32.8	0	1
67	sub-feta036	Neurotypical	22.7	1	1
68	sub-feta037	Neurotypical	23.4	0	1
69	sub-feta038	Neurotypical	26.9	1	1
70	sub-feta039	Neurotypical	24.3	0	1
71	sub-feta081	Pathological	30.2	1	1
72	sub-feta084	Pathological	28.7	0	1
73	sub-feta085	Pathological	22.9	0	1
74	sub-feta086	Pathological	25.8	0	1
75	sub-feta087	Pathological	27.5	0	1
76	sub-feta088	Neurotypical	21.2	1	1
77	sub-feta089	Neurotypical	28.6	0	1
78	sub-feta090	Neurotypical	32.1	1	1
	Total simulated			29	70

[1] Payette, K. & Jakab, A. Fetal tissue annotation challenge - feta miccai 2021, 10.7303/SYN25649159 (2021). [2]Fidon, L. et al. Label-set loss functions for partial supervision: Application to fetal brain 3D MRI parcellation; 10.5281/zenodo.6405632 (2021).
